# A Comparison of cVEMP and VNG Examination Results Between Adults and Children with a History of Vertigo

**DOI:** 10.3390/jcm14072222

**Published:** 2025-03-25

**Authors:** Anna Waśniewska-Włodarczyk, Oskar Rosiak, Renata Pepaś, Filip Wróbel, Wiesław Konopka

**Affiliations:** Department of Otolaryngology, Polish Mother’s Memorial Hospital Research Institute, 93-338 Lodz, Polandrenatapepas@interia.eu (R.P.); filip.wrobel@stud.umed.lodz.pl (F.W.); wieslaw.konopka@umed.lodz.pl (W.K.)

**Keywords:** videonystagmography, VNG, cVEMP, age, vertigo, adult, children, IFO, COR, VOR

## Abstract

**Background/Objectives:** Dizziness and vertigo are common symptoms. Vertigo, caused by vestibular deficit, is usually diagnosed with videonystagmography (VNG) and cervical vestibular evoked myogenic potential (cVEMP). Normative values of these examinations have been established for adults; however, the impact of age is still uncertain. This study aimed to compare the results of VNG and cVEMP between adults and children. **Methods:** We analyzed and compared the results of VNG and cVEMP in 119 patients (35 adults and 84 children.) **Results:** Statistically significant differences were observed between adults and children in the amplitude of the cVEMP examination. In the subgroup analysis by age, we also noticed differences in VNG examination in nystagmus induced by rotation in patients with peripheral vertigo and in IFO (index fixation test) and VOR (vestibulo-ocular reflex) in patients with non-peripheral vertigo. **Conclusions:** This study confirms that there are differences in vestibular examination results between children and adults. However, the exact impact of age on each part of the vestibular examination requires further investigation.

## 1. Introduction

Dizziness and vertigo are common symptoms in children. Dizziness refers to various sensations, including floating, disequilibrium, disorientation, and a sense of imminent fall. Vertigo is characterized as a motion illusion, such as the feeling of spinning or movement. These symptoms may indicate dysfunction in the visual, somatosensory, or vestibular systems or impairment along the entire pathway [1]. Vestibular deficits in childhood may lead to forced head positions, coordination disorders, and delayed control of posture [2]. According to Davitt et al. [3], 5.3% of pediatric patients complain about vertigo, whereas according to Neuhauser [4], dizziness (including vertigo) in adults occurs in 15–35% of the population. In a study reported by Bhandari et al. [5], the most common disorders are vestibular migraine (63.39%), benign paroxysmal vertigo (BPPV) (24.48%), central vestibulopathy (14.1%), peripheral vestibulopathy (17.42%), and other causes (6.19%).

Since it is difficult to obtain an exact medical history from a child (those under 18 years old), making a correct diagnosis in pediatric patients is especially challenging. Children often struggle to articulate the duration of a vertigo attack, what triggers it, or the specific sensations they experience during the episode. Their descriptions may be vague or limited due to their age and understanding. A thorough clinical examination, including vestibular and oculomotor assessment, leads to a correct diagnosis [6]. The clinical examination should include an oculomotor evaluation with Frenzel goggles, postural tests including tandem stance and one-leg stance, and gait evaluation. Further diagnostic tests include specific objective evaluations of particular receptors of the inner ear, such as the saccule, utricle, and semicircular canals.

There are multiple methods for diagnosing vestibular dysfunction. Quantitative tests include cervical vestibular evoked myogenic potential (cVEMP), ocular vestibular evoked myogenic potential (oVEMP), video head impulse test (vHIT), the caloric test, and the rotatory chair test. cVEMP tests the vestibulocollic reflex, evaluating saccular function and the inferior vestibular nerve by measuring inhibition of the ipsilateral sternocleidomastoid muscle (SCM) [7]. cVEMP does not require children to be in the dark, the examination time is short, and it does not lead to dizziness. However, cVEMP requires placing electrodes on the neck and face, so children younger than 10 years old may feel fear or discomfort.

VNG consists of multiple tests that evaluate the vestibular system, helping to differentiate between peripheral and central disorders and localize lesions by assessing nystagmus. In principle, the test depends on the examination and quantitative evaluation of the movement of the pupil in the dark, measured by infrared cameras mounted inside the VNG goggles during different tests. It is usually accompanied by a caloric test, which specifically tests the reaction of the lateral semicircular canal to low-frequency stimulus. Although a caloric test is especially significant in diagnosing vertigo due to ear-specific information, children find this test frightening because of the water irrigation, which may be unpleasant, induce dizziness, and, in many cases, requires them to lie still for a couple of minutes, which can be difficult. The rotatory chair test is an easy VNG test for children because it is fast, and children may sit on their parent’s lap. Nevertheless, some parts of the rotatory chair test require darkness, the goggles are often too big for the child, and it requires the child’s attention [7].

Although VNG and cVEMP testing are well-established methods in adult vestibular evaluation, their utility in the pediatric population is limited. The probable maturational effect indicates that adult normative data for VNG examination might not be suitable for children [8,9]. According to Pyda-Dulewicz et al. [10], at the age of 4 or 5, a child’s nervous system typically reaches sufficient development to diagnose vertigo using adult normative values [10]. Values of gain for IFO (index fixation test), COR (cervico-ocular reflex), and VOR (vestibulo-ocular reflex) may vary slightly depending on the type of equipment and software.

This retrospective study aims to assess and compare the results of cVEMP and VNG in adults and children. We formulated the following research questions and hypotheses:

- Does age affect the values of the asymmetry ratio in cVEMP?

**Hypothesis 1.**  *Age does not affect the values of the asymmetry ratio in cVEMP.*

- Does age affect the values of gain of COR, IFO, and VOR in VNG tests?

**Hypothesis 2.**  *There is a difference in gain scores between adults and children.*

## 2. Materials and Methods

In this study, we analyzed the results of VNG and cVEMP examinations in 119 patients hospitalized between 2020 and 2024 at the Department of Otolaryngology, Polish Mother’s Memorial Hospital Research Institute. The inclusion criteria were a history of vertigo and available medical records detailing otolaryngological and neurological examinations, including vestibular tests (cVEMP, VNG). The diagnosis of peripheral or non-peripheral vertigo was based on the available medical records detailing the otolaryngological and neurological examination, including vestibular tests (cVEMP, VNG).

### 2.1. cVEMP

cVEMP registration was performed using an Intelligent Hearing Systems SmartEP (version 3.94) device for electrophysiological studies. cVEMP responses were recorded from the sternocleidomastoid muscle using an 88 dB nHL click and a 500 Hz tone burst. In the quantitative analysis, the peak amplitude was measured from the top of p13 to the top of n23. An inter-ear amplitude difference exceeding 35% was considered abnormal.

### 2.2. VNG

The VNG tests were conducted using Audical’s Framiral system (version 1.6.39), equipped with VNG goggles featuring infrared cameras and fixation diodes, and a rotation chair. The full VNG assessment included spontaneous and gaze-evoked nystagmus, saccades, optokinesis, and caloric testing.

#### 2.2.1. BURST

During the BURST protocol, the patient underwent horizontal rotations of increasing and decreasing amplitude with a frequency of 0.1 Hz and a maximum velocity (Vmax) of 50°/s. Each trial lasted 20 s. The BURST protocol consisted of different types of tests, such as VOR, COR, and IFO. During VOR testing, patients closed their eyes while the chair moved (nystagmus of labyrinthine origin). In COR testing, patients also closed their eyes, but their heads remained motionless, supported by the clinician (nystagmus of cervical origin). During IFO testing, patients kept their eyes open and focused on a colorful object in their hands. Normative gain values were assumed as follows: IFO < 0.2, VOR > 0.5, and COR < 0.4, based on the Framiral software (version 1.6.39) reference standards.

#### 2.2.2. Caloric Test

The caloric test was conducted with water at temperatures of 30 °C and 44 °C. Unilateral weakness (UW) was determined by comparing the responses of the right and left labyrinths based on the formula for directional preponderance ([CR + WR] − [CL + WL]/(CL + WL + CR + WR) × 100%. A result of >25% indicated a unilateral peripheral weakness on the side of the weaker response.

#### 2.2.3. Nystagmus Induced by Rotation

In the nystagmus induced by rotation (NIR) test, the chair was rotated at a speed of 90–120°/s, with three clockwise rotations followed by a rapid stop and three counterclockwise rotations followed by a rapid stop. The direction predominance was counted as a comparison of velocities. An asymmetry >25% between rightward and leftward eye movements was considered abnormal. Data on diagnoses were also collected, distinguishing between peripheral and non-peripheral vertigo.

#### 2.2.4. Statistical Analysis

The data were analyzed using Statistica (ver. 13.1). To determine the normality of the distribution of variables, a Shapiro–Wilk test was used; continuous variables were expressed as a minimum, maximum, and mean ± standard deviation. For non-normal distributions, the median and interquartile ratios were analyzed. Categorical data were described with absolute frequencies and percentages. Depending on the distribution of the variables, comparisons between groups were performed using Student’s *t*-test (or nonparametric Mann–Whitney U test) and Fisher’s exact test (or χ^2^ test). When more than two groups were analyzed, a one-way ANOVA followed by the Tukey post hoc test (or, for nonparametric data, the Kruskal–Wallis test followed by Dunn’s post hoc test, depending on the distribution of variables) was performed. A value of *p* < 0.05 was considered statistically significant.

## 3. Results

**Figure 1:**
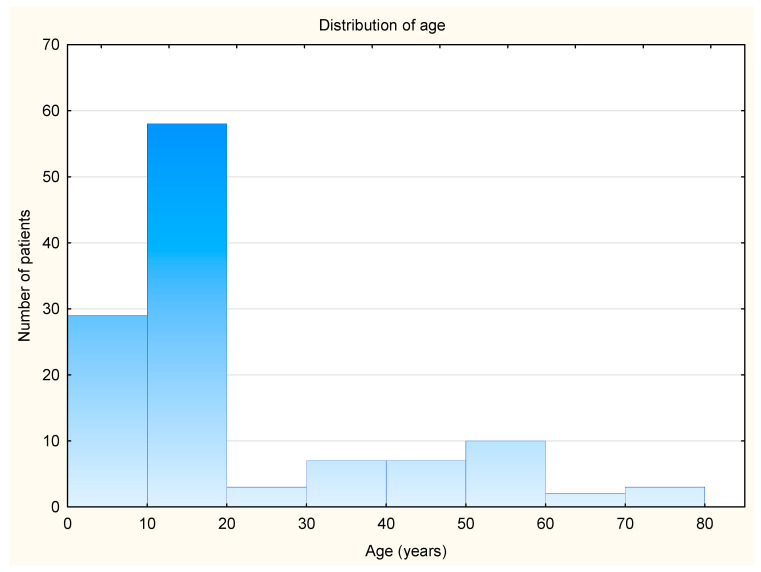
Age distribution of patients included in this study.

This study is a retrospective analysis involving 119 patients, comprising 49 men (41.18%) and 70 women (58.82%). Among them, 35 patients (29.41%) were adults, while 84 patients (70.59%) were children younger than 18 years old. The mean age of the patients enrolled in this study was 21.76 ± 17.94 years. The youngest patient was 2.8 years old, while the oldest was 74.3 years old. The age distribution is shown in Figure 1.

The values obtained from the examinations are detailed in Table 1. Results from cVEMP were obtained from 82 patients, BURST and NIR from 105 patients, and the caloric test from 53 patients. A total of 29 patients were diagnosed with peripheral vertigo (e.g., BPPV, vestibular hypofunction, concussion of the labyrinth, vestibular paroxysmia, vestibular areflexia); among them, 17 were children, and 12 were adults. A total of 90 patients were diagnosed with non-peripheral vertigo, of whom 48 were children and 42 were adults. In the cVEMP examination, the only significant difference in results between adults and children was noticed in amplitude in patients with non-peripheral vertigo (*p* = 0.043) (Table 2, Table 3 and Table 4). The mean VOR gain value in patients with non-peripheral vertigo was significantly higher in children than in adults (*p* = 0.042, Table 2). In patients with peripheral vertigo, the mean NIR value was significantly higher in children than in adults (*p* = 0.032, Table 2). No patients with peripheral vertigo were younger than 6 years old (Table 3 and Table 5).

**Figure 2:**
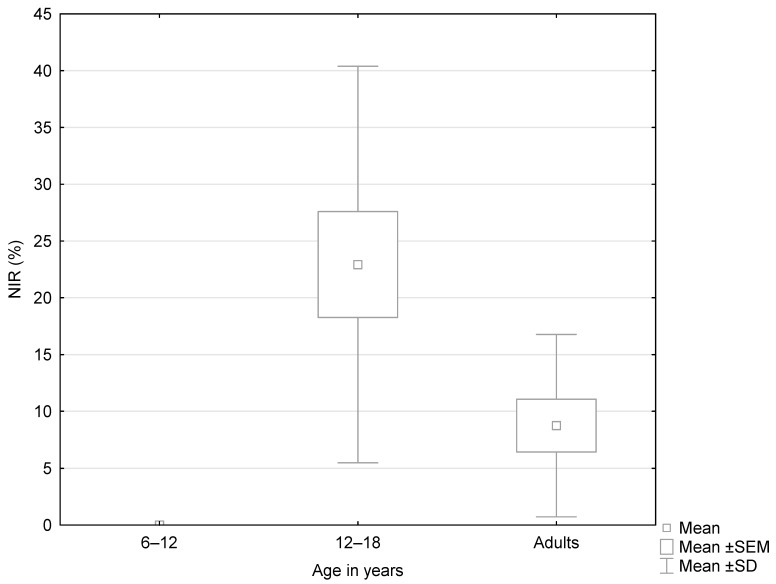
Box-and-whisker plots representing the results of NIR (%) in patients with peripheral vertigo depending on the age group.

**Figure 3:**
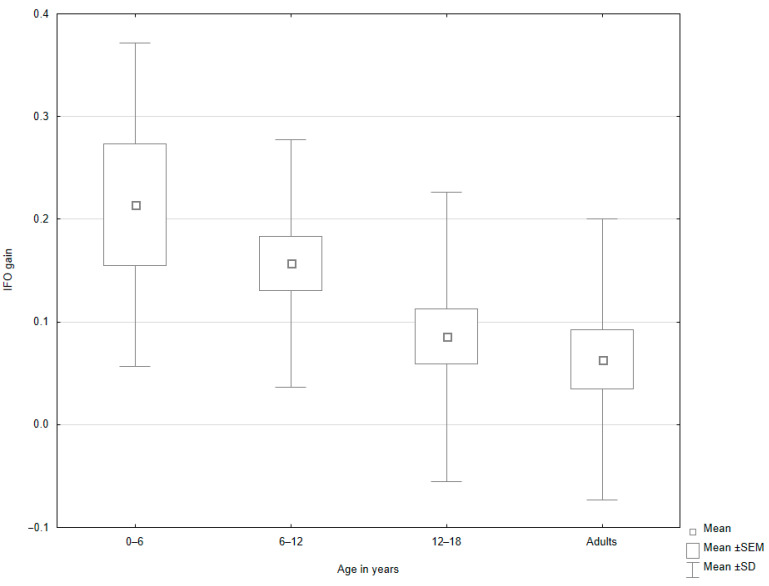
Box-and-whisker plots representing the results of IFO gain in patients with non-peripheral vertigo, depending on the age group.

**Figure 4:**
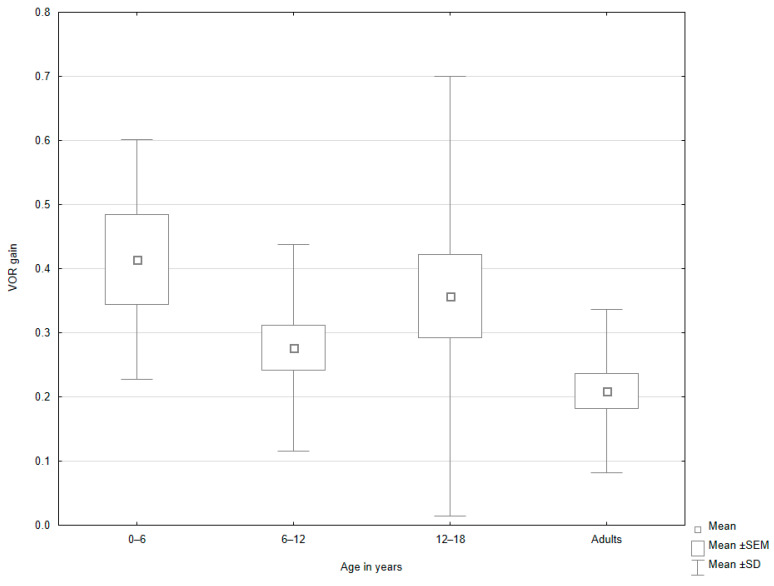
Box-and-whisker plots representing the results of VOR gain in patients with non-peripheral vertigo, depending on the age group.

In the subgroup analysis, age significantly influenced the mean NIR value among patients with peripheral vertigo (*p* = 0.047, Figure 2, Table 5). The post hoc test showed that the NIR value was higher in the group of patients aged 12–18 years old than in adults. Age was also a significant factor for the mean IFO value among patients with non-peripheral vertigo (*p* = 0.002; Table 6). In the following post hoc evaluation, the group aged 6–12 years old was characterized by a higher mean IFO value when compared to adults (Figure 3). Moreover, significant differences were observed in the mean VOR value between age subgroups in patients with non-peripheral vertigo (Table 6). The post hoc analysis revealed that the mean VOR value was higher in children under 6 years old than in adults (*p* = 0.046; Figure 4).

## 4. Discussion

The primary objectives of this study were to verify if there are differences in data between children and adults with a history of vertigo in (1) cVEMP and (2) VNG examinations.

In this study, we have demonstrated that cVEMP and VNG can be easily conducted at any age. Martens et al. [11] suggested using cVEMP to evaluate labyrinth dysfunction as a screening method in infants as young as 6 months old. We did not observe a significant influence of age on the asymmetry ratio of cVEMP in both groups of patients with peripheral and non-peripheral vertigo. However, we noticed a statistically significant difference between children and adults with non-peripheral vertigo in p13–n23 amplitude. Similar findings were reported by Lee et al. [12], who examined 97 patients aged 12–77 years old. Although they noticed a significant positive correlation between p13 and n23 latency and age, they did not observe a correlation between age and the asymmetry ratio.

In adults, Maheu et al. [13] compared younger patients (mean age 22.79 years old) and older patients (mean age 69.0 years old). The raw amplitude asymmetry ratios of cVEMP did not differentiate significantly between groups (*p* = 0.726). McCaslin et al. [14] conducted a cVEMP examination on 97 volunteers aged 5–67. Volunteers were divided into three groups according to age: pediatric (5–17 years old), young adults (19–40 years old), and older adults (41–70 years old). The statistical analysis showed that the significant paired comparisons were between pediatric and older adult groups only (*p* = 0.001).

As previous studies have shown, cVEMP results are affected by multiple factors. There might be a potential influence of head position and contraction of the sternocleidomastoid muscle, type of acoustic stimuli, or ethnicity [15]. In the study reported by Chang et al. [16], there was a positive correlation between p1 latency and neck length. Moreover, Lim et al. [15] reported a positive correlation between p1 and n1 latencies and a negative correlation between p1-n1 amplitude and neck length. It suggests that a difference in cVEMP results between children and adults may be caused not by age but by neck length. Notably, it is challenging to compare different studies of cVEMP asymmetry due to differences in recording characteristics and stimuli [14].

Rotational chair testing is a more sensitive examination than caloric testing in diagnosing peripheral vestibulopathy. Thus, rotational chair testing is often used as a screening test in patients who do not tolerate caloric testing, mainly among the pediatric population. During diagnosis, normative gains of rotational chair testing are provided by the equipment manual instruction. However, manufacturers usually obtain their data on a small number of test subjects, and the age of the subjects remains unknown [17].

Previous studies are not consistent on whether age affects VOR gain and other parts of the VNG examination. Valente [8] investigated 60 healthy children divided into two age groups: 3–6 years old and 9–11 years old. They did not find a significant correlation between these two age groups and VOR gain. Charpiot et al. [18] compared three groups of healthy patients: aged 6–8 years, 9–10 years, and 11–12 years. They found VOR gain lower in groups aged 9–10 years old and 11–12 years old compared to the group aged 6–8 years old.

In 2015, Chan et al. [17] presented normative data for the rotational chair stratified by age. Their data were collected from an examination of 100 patients aged from 6 to 78 years old. They divided patients into five subgroups according to age (I 6–12; II 13–17; III 18–30; IV 31–50; V >50 years old). They observed an inverse trend between VOR gain and age. Moreover, they compared their results with manufacturers’ normative data. Most of the VOR gain of adults fell within the specified limits; however, the mean value for group I exceeded the manufacturer’s upper limit by two SDs. Furthermore, the results obtained by group I were significantly different compared to all other groups.

According to Maes et al. [19], the existing literature data considering the velocity step test are conflicting, which may be caused by different velocity, acceleration, and deceleration parameters. They analyzed 80 healthy patients aged 18–80 years old and divided them into two groups: over 50 years old and under 50 years old. They noticed subtle differences in rotational chair tests and caloric tests between the groups but highlighted a strong correlation between age and VEMP results.

In this study, adults with non-peripheral vertigo had a significantly lower VOR gain compared to children. Conversely, children with peripheral vertigo are characterized by higher velocity of nystagmus in the NIR test than adults. This finding indicates that younger individuals may exhibit stronger or more pronounced vestibular responses in peripheral vertigo cases, which could be attributed to differences in neural plasticity, compensatory mechanisms, or anatomical factors. Moreover, in the group of patients with non-peripheral vertigo, we noticed a significant negative correlation between age and IFO and VOR gain. According to Śpiewak et al. [20], the sensitivity of the vestibular organ to kinetic stimuli decreases with age.

Caloric testing is a significant part of the vestibular examination; however, it is especially challenging in younger children. We were able to perform caloric tests only in 33 children, where the youngest patient was 5.8 years old. The responses to caloric testing are believed to mature between 6 and 12 months of age, and the accuracy of the examination improves as the child’s weight increases. Andrieu-Guitrancourt et al. [21] investigated 140 healthy children aged 2–10 years old with air caloric tests. The authors reported that in response to air stimuli, the magnitude of the slow-phase velocities negatively correlated with age. Melagrana et al. [22] compared caloric responses between 42 healthy children and 57 healthy adults. Their data showed that the SDs for unilateral weakness and directional preponderance in children were higher than in adults.

In this study, UW values were similar between adults and children in both groups, with peripheral and non-peripheral vertigo. Additionally, we did not notice a significant difference between age subgroups. Our findings indicate that further comparative studies are warranted to investigate UW. Future research should focus on larger, more heterogeneous patient cohorts and employ advanced diagnostic methodologies to assess potential influencing factors. Variables such as vertigo duration, severity, etiology, and the presence of comorbid vestibular pathologies should be systematically analyzed to determine their impact on UW values.

## 5. Limitations

Vestibular testing is influenced by factors such as age, mental development, and the ability of the subject to follow instructions. Tests that are common and easy to perform in adults, like caloric testing, can be challenging in children. In the children group, a full VNG examination was not always conducted due to a lack of cooperation. This limitation also contributed to our decision to categorize patients into only two broad groups: “peripheral” and “non-peripheral”. The study group is heterogeneous, and some diagnoses are singular. As a result, we were unable to analyze the examination results according to specific diagnoses. Additionally, vestibular examinations are prone to artifacts, such as patient movement or equipment issues, which can affect the reliability of results. Another limitation is the unequal sample size across different subgroups, which can affect the accuracy and generalizability of the findings. Moreover, in this study, we did not prepare age-matched control groups of healthy volunteers due to the retrospective nature of the study.

## 6. Conclusions

This study confirms that there are some differences in vestibular examination results between children and adults. In the cVEMP examination, the only significant difference in results between adults and children was noticed in amplitude in patients with non-peripheral vertigo. Differences in vestibular examinations were especially noticeable in the rotational chair test. IFO gain and VOR gain were negatively correlated with age. Due to the limitations of this study, the data are insufficient to support our hypothesis. The exact impact of age on each part of the vestibular examination remains unknown. Our results highlight the need for further comparative studies to establish new normative data for vestibular examination in children.

## Figures and Tables

**Table 1:** Mean values.

Type of Examination	Parameter	Mean	Minimum	Maximum	Standard Deviation ±
cVEMP	Asymmetry ratio–click (%)	23.78726	0.238500	78.98490	20.25237
	Latency p13–click (ms)	14.75920	1.89604	10.10000	19.9500
	Amplitude p13–click (ms)	59.31541	55.07040	3.83495	288.5702
	Asymmetry ratio–500 Hz (%)	23.33700	0.470400	78.98490	18.10887
	Latency p13–500Hz (ms)	13.06383	2.35600	5.80000	17.5000
	Amplitude–500 Hz (ms)	43.42098	35.84841	2.75550	188.2980
VNG	IFO gain	0.10265	0.000000	0.80000	0.14421
	VOR gain	0.28850	0.000000	1.80000	0.22825
	COR gain	0.07522	0.000000	0.80000	0.14176
Rotational test	NIR (%)	13.48673	0.000000	55.00000	12.28829
Caloric test	UW(%)	15.33898	0.000000	78.00000	16.34289

**Table 2:** Differences between adults and children with peripheral and non-peripheral vertigo. * Denotes statistically significant differences.

Group of Patients Type of Examination		Non-Peripheral Vertigo, Children, Mean	Non-Peripheral Vertigo, Adults, Mean	*p*	Peripheral Vertigo, Children, Mean	Peripheral Vertigo, Adults, Mean	*p*
cVEMP	Asymmetry ratio–click (%)	23.10	21.37	0.76	27.84	31.18	0.70
	Latency p13–click (ms)	14.78	14.66	0.84	15.02	14.40	0.51
	Amplitude p13–click (ms)	70.86	36.67	0.043 *	53.3	39.75	0.06
	Asymmetry ratio–500 Hz (%)	19.18	19.39	0.97	32.32	28.11	0.68
	Latency p13–500Hz (ms)	12.67	13.76	0.14	13.84	13.10	0.37
	Amplitude p13–500 Hz (ms)	48.85	32.01	0.25	41.93	31.77	0.42
VNG	IFO gain	0.13	0.06	0.07	0.15	0.03	0.08
	VOR gain	0.33	0.21	0.042 *	0.33	0.23	0.15
	COR gain	0.09	0.06	0.35	0.07	0.03	0.44
Rotational test	NIR (%)	12.34	13.64	0.65	21.40	8.75	0.032 *
Caloric test	UW (%)	7.00	7.08	0.97	30.00	30.29	0.98

**Table 3:** Differences in age subgroups in cVEMP examination in patients with peripheral vertigo. Data were analyzed using a one-way ANOVA or Kruskal–Wallis test, depending on the distribution of variables.

cVEMP Results Age Group (Number of Patients)	Asymmetry Ratio-500 Hz	Latency p13	Amplitude p13/n23	Asymmetry Ratio Click	Latency p13	Amplitude p13/n23
0–6 years old (*n* = 0)	-	-	-	-	-	-
6–12 years old (*n* = 2)	9.31	14.70	17.00	58.52	12.80	12.00
12–18 years old (*n* = 15)	29.26	15.05	13.92	30.31	13.91	14.14
Adult (*n* = 12)	31.18	14.40	8.67	28.11	13.10	11.50
*p*	0.57	0.80	0.15	0.58	0.59	0.68

**Table 4:** Differences in age subgroups in cVEMP examination in patients with non-peripheral vertigo. Data were analyzed using a one-way ANOVA or Kruskal–Wallis test, depending on the distribution of variables.

cVEMP Results Age Group (Number of Patients)	Asymmetry Ratio 500 Hz	Latency	Amplitude	Asymmetry Ratio Click	Latency	Amplitude
0–6 years old (*n* = 9)	15.7	15.41	24.57	32.5	39.86	31.00
6–12 years old (*n* = 26)	21.8	14.05	39.16	14.03	28.77	41.68
12–18 years old (*n* = 32)	26.8	15.17	34.75	20.18	35.12	33.38
Adult (*n* = 23)	21.37	14.66	23.57	19.39	42.14	29.50
*p*	0.52	0.16	0.06	0.2	0.23	0.27

**Table 5:** Differences in age subgroups in VNG examination in patients with peripheral vertigo. Data were analyzed using a one-way ANOVA followed by the Tukey post hoc test or Kruskal–Wallis test followed by Dunn’s post hoc test, depending on the distribution of variables. * Denotes statistically significant differences.

VNG Results Age Group (Number of Patients)	IFO Gain	VOR Gain	COR Gain	NIR (%)	UW (%)
0–6 years old (*n* = 0)	-	-	-	-	-
6–12 years old (*n* = 2)	0.80	0.10	0.0	0.0	23.00
12–18 years old (*n* = 15)	0.10	0.35	0.08	22.93	31.40
Adult (*n* = 12)	0.033	0.26	0.03	8.75	30.29
*p*	0.66	0.21	0.80	0.047 *	0.89

**Table 6:** Differences in age subgroups in VNG examination in patients with non-peripheral vertigo. Data were analyzed using a one-way ANOVA followed by the Tukey post hoc test or Kruskal–Wallis test followed by Dunn’s post hoc test, depending on the distribution of variables. * Denotes statistically significant differences.

VNG Results Age Group (Number of Patients)	IFO Gain	VOR Gain	COR Gain	NIR	UW
0–6 years old (*n* = 9)	0.21	0.41	0.06	16.86	9.00
6–12 years old (*n* = 26)	0.16	0.28	0.10	13.19	6.67
12–18 years old (*n* = 32)	0.09	0.36	0.10	10.57	7.09
Adult (*n* = 23)	0.06	0.21	0.06	13.64	7.08
*p*	0.002 *	0.046 *	0.43	0.46	0.9

## Data Availability

The datasets generated and/or analyzed during the current study are not publicly available due the state of the hospital but are available from the corresponding author on reasonable request.

## Abbreviations

The following abbreviations are used in this manuscript:

COR	Cervico-ocular reflex
cVEMP	Cervical vestibular evoked myogenic potential
IFO	Index fixation test
NIR	Nystagmus induced by rotation
UW	Unilateral weakness
VNG	Videonostagmography
VOR	Vestibulo-ocular reflex
